# Mechanisms of Panax ginseng action as an antidepressant

**DOI:** 10.1111/cpr.12696

**Published:** 2019-10-10

**Authors:** Yang Jin, Ranji Cui, Lihong Zhao, Jie Fan, Bingjin Li

**Affiliations:** ^1^ Jilin Provincial Key Laboratory on Molecular and Chemical Genetic Second Hospital of Jilin University Changchun China

**Keywords:** active ingredients, antidepressant mechanism, depression, neuron, Panax ginseng

## Abstract

**Objectives:**

Panax ginseng, a well‐known traditional Chinese medicine with multiple pharmacological activities, plays a crucial role in modulating mood disorders. Several recent studies have identified an underlying role of Panax ginseng in the prevention and treatment of depression. However, the cellular and molecular mechanisms remain unclear.

**Materials and Methods:**

In this review, we summarized the recent progress of antidepressant effects and underlying mechanisms of Panax ginseng and its representative herbal formulae.

**Results:**

The molecular and cellular mechanisms of Panax ginseng and its herbal formulae include modulating monoamine neurotransmitter system, upregulating the expression of neurotrophic factors, regulating the function of HPA axis, and anti‐inflammatory action.

**Conclusions:**

Therefore, this review may provide theoretical bases and clinical applications for the treatment of depression by Panax ginseng and its representative herbal formulae.

## 1 INTRODUCTION

Depression, academically known as major depressive disorder (MDD), is a prevalent mental disorder which is characterized by a pervasive and sustained low mood, low interest, low motivation and even suicidal tendencies.^1^ MDD reduces the quality of life for numerous people all around the world.^2^, ^3^ Nevertheless, little is known about the underlying pathogenesis and aetiology of depression. Widely accepted molecular theories of depression include monoaminergic hypothesis, monoaminergic receptor hypothesis, neurotrophic hypothesis, the hypothesis of neuroplasticity, neuroendocrine and neuroimmune hypothesis.^4^, ^5^, ^6^, ^7^, ^8^, ^9^, ^10^

For the treatment of depression, current clinical therapy mainly depends on traditional antidepressants. However, traditional antidepressants have a limitation in long onset time, high relapse rates and severe side effects, such as cognitive dysfunction, sexual dysfunction and sleep disorders.^11^, ^12^, ^13^ Besides, depression poses a huge physiological and economic burden on patients with depression. Therefore, it is critically important to develop efficient and safe drugs for depression treatment. Recent studies have revealed that traditional herbal medicines may offer promising alternatives for depression treatment with high safety and tolerance.^14^, ^15^, ^16^ Panax ginseng Meyer is a well‐known traditional medicinal herb which has been used as a vital energy reinforcing agent with a long history in Chinese medicine theory. Ginseng exerts diversified biological activities, for instance, anti‐tumour, anti‐inflammatory, anti‐oxidation, inhibition of cell apoptosis and neuroprotective effects.^17^ Recently, some studies have shown that ginseng plays a significant role in depression and may act as a potential antidepressant. Moreover, ginseng can be taken for a long duration with a high safety profile and less adverse reactions. Although some relevant mechanisms have been discussed, studies on the antidepressant‐like effects of ginseng are still in their infancy.

In the theory of traditional Chinese medicine, depression is a general term for a class of diseases and syndromes caused by emotional impairments and dysfunctions of Qi and blood. Deficiency of Qi and blood leads to disorder of Qi, damaging the Qi of viscera and affecting the normal function of viscera. Qi was considered as a specific substance which is essential to survival for the human body, and it is also the general name of the functional activities of human organs. Ginseng is deemed to play a role of invigorating vital energy by sufficiently tonifying Qi of spleen, lung, heart and kidney. Meanwhile, Qi promotes blood production. Ginseng makes the body produce blood, smooth blood vessels and restore Qi and blood, and subsequently nourish the heart and calm the mind via tonifying Qi. In the present paper, we systematically reviewed the likely underlying mechanisms of antidepressant effects of ginseng and its herbal formulae and pave a way for the further study of ginseng in the treatment of depression.

## 2 GINSENG AND ITS ACTIVE INGREDIENTS

Ginseng, a precious herb in traditional Chinese medicine, has a long history of medicinal use in the improvement of mental state and modulation of neurological diseases, such as insomnia, depression, anxiety and neurasthenia. It has been honoured as "the king of herbs" and was listed as the top grade in Shennong Materia Medica. According to different origins, there are basically Panax ginseng, Panax quinquefolium L. and Panax notoginseng. They are also the most extensively studied species. Panax ginseng, which has the greatest tonic effect, is mainly cultivated in northeast China, Korea, Japan and eastern Russia. It is best for people who are physically weak and infirm, preferably taken in winter. Panax quinquefolium L., also known as American ginseng, is mainly produced in the United States, Canada and other countries. It is the mildest in nature and suitable for most people and all seasons. Panax notoginseng is only grown in parts of China, including Jilin, Guangxi and Yunnan.^18^ In addition, according to the different processing methods, ginseng can also be divided into white ginseng, sugar ginseng and red ginseng (Table 1). Ginseng has attracted the attention of many researchers all over the world because of its wide range of pharmacological effects and medical application efficacy. Modern studies show that ginseng contains a large number of active ingredients, including ginsenosides, ginseng polypeptides, ginseng polysaccharides and so on (Table 2). Among them, ginsenosides are considered to be major active ingredients of ginseng physiological activity, which have impacts on the nervous system, cardiovascular system, immune system and so on.^19^ On the basis of different chemical structures of their aglycons, ginsenosides can be classified into three main types: protopanaxadiols (PPDs), protopanaxatriols (PPTs) and oleanolic acids. PPD‐type includes ginsenosides Rb1, Rb2, Rc and Rd, while PPT‐type includes ginsenosides Rg1, Rg2, Rf and Re. Besides, Ro is the main oleanolic acid‐type ginsenoside. The structural formulae of ginsenosides with antidepressant effects are presented in Table 3. Ginseng polysaccharides, as another key bioactive component of ginseng, also have many biological activities, such as immune regulation, anti‐cancer, anti‐depression and anti‐oxidation. It is a kind of polymer dextran, composed of ginseng neutral sugars and ginseng acid pectin. Between them, the depression studies of ginseng polysaccharides mainly focus on the acidic sugars‐pectin part of ginseng. The antidepressant effects of ginseng and its extracts have been confirmed by a wide range of clinical applications and experimental studies. Kim et al^20^ reported that several kinds of processed ginsengs all could improve depression‐like behaviours in forced swimming test (FST). Administration of wild ginseng extracts strongly reduced depression‐like behaviours induced by morphine withdrawal through regulating the hypothalamus corticotrophin‐releasing factor and neuropeptide Y, which indicated the possible antidepressant‐like effects of wild ginseng.^21^

**Table 1:** Classification of ginseng according to different processing methods

Category	Common processing methods
White ginseng	Made of fresh ginseng by boiling in boiling water for a moment and drying
Red ginseng	Made of fresh ginseng by steaming at high temperature and drying
Sugar ginseng	Made of fresh ginseng by needle‐piercing, soaking in sugar water and drying
Radix ginseng cruda	Made of fresh ginseng by natural drying or artificial drying

**Table 2:** Complex active ingredients of Panax ginseng

Category	Active ingredients	Effects
Ginsenosides	Protopanaxadiols: Ra1, Ra2, Ra3, Rb1, Rb2, Rb3, Rc, Rd, Rg3, Rh2 Protopanaxatriols: Rg1, Rg2, Re, Rf, Rh1, Rh3 oleanolic acid: Ro	Anti‐depression, anti‐tumour, anti‐ageing, anti‐ischaemic brain injury, immunomodulation, CNS regulation
Ginseng polysaccharides	Monosaccharide: glucose, galactose, arabinose, rhamnose, galacturonic acid, mannose etc Disaccharide: sucrose, maltose, locust, etc Polysaccharide: ginseng trisaccharide and ginseng tetrasaccharide	Immunomodulation, anti‐tumour, inhibition of liver injury, hypoglycaemic activity
Ginseng polypeptides	Oligopeptide I, II, II, IV	Hypolipidemic and hepatic glycogen‐lowering
Volatile oils	Sesquiterpenes, β‐elemene, panaxynol panaxydol, panaxytriol, ginsenyne, panasinsene, etc	Bacteriostasis and improvement of myocardial ischaemic injury
Polyacetylenes	Panaxydol, panaxytriol	Anti‐tumour and anti‐leukaemia
Organic acids and esters	Citric acid, isocitric acid, fumaric acid, linoleic acid, malic acid, succinic acid, tartaric acid, panax acid, triglyceride, palmitic acid, etc	Anti‐oxidation
Others	Microelements, vitamins, alkaloids, lignins	Regulation of growth, metabolism and immune function

**Table 3:** Structural formulae of ginsenosides with antidepressant effects

Ginsenoside	Molecular formula	Chemistry structural formula
20(S)‐Ginsenoside‐Rb1	C_54_H_92_O_23_	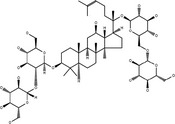
20(S)‐Ginsenoside‐Rg1	C_42_H_72_O_14_	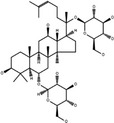
20(S)‐Ginsenoside‐Rg2	C_42_H_72_O_13_	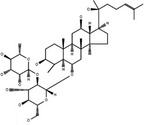
20(S)‐Ginsenoside‐Rg3	C_42_H_72_O_13_	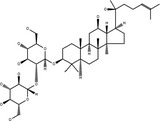
20(S)‐Ginsenoside‐Rg5	C_42_H_70_O_12_	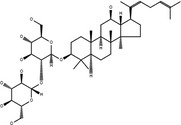
20(S)‐Ginsenoside‐Rh1	C_36_H_62_O_9_	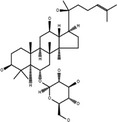

## 3 ANTIDEPRESSANT MECHANISMS OF PANAX GINSENG

### 3.1 Effect on monoamine neurotransmitters

In the past years, monoamine neurotransmitters have become a focus on the pathophysiology of depression. It is reported that depression is caused by a decline in the function of monoamine transmitters, such as serotonin (5‐HT), noradrenaline (NA) and dopamine (DA). Indeed, reduction of plasma 5‐HT level has been observed in depressed patients resulting from alcohol withdrawal.^22^ At present, antidepressants based on monoamine transmitters are still the first‐line antidepressants. Through the metabolomics in serum, urine and cerebral tissue of chronic unpredictable mild stress (CUMS) rats, ginsenosides were found to mitigate changes in amino acid neurotransmitters and monoamine neurotransmitters.^23^ Similarly, pre‐treatment with ginseng fruit saponins could significantly increase the 5‐HT levels in both serum and platelets but not reverse the decline of 5‐HT level in the brain induced by an experimental comorbidity model of myocardial infarction (Akbay's model) and depression (modified forced swimming model). Besides, ginseng fruit saponins could decrease the 5‐HT_2A_R expression in both brain and platelets.^24^, ^25^ Ginsenoside Rb1, active component of the ginseng root, significantly upregulated the 5‐HT, 5‐HIAA, NA and DA levels in the brain of CUMS rats and also exhibited a synergistic effect when combined with caffeine and fluoxetine.^26^ Besides, ginsenoside Rb1 and its metabolite compound K acted as antidepressants by regulating 5‐HT_2A_ receptors, which was demonstrated by blockade action of ritanserin pre‐treatment.^27^ Gintonin, an active constituent of the Panax ginseng extract, could improve depression‐related symptoms in mice with alcohol withdrawal through elevating the level of plasma 5‐HT which was released from intestinal enterochromaffin cells.^28^ Chronic treatment of total saponins extracted from Panax notoginseng also could improve the rats depression‐like behaviours in chronic mild stress (CMS) mice via increasing the levels of 5‐HT, DA and NA and decreasing cytoplasmic free intracellular Ca^2+^ concentration.^29^ Other neurotransmitters, such as acetylcholine and glutamate, also have been found to be strongly associated with depression.

### 3.2 Effect on hypothalamic‐pituitary‐adrenal axis

The neuroendocrine hypothesis points out that dysfunction of the endocrine system is the main cause of the occurrence of depression. Hyperactivity of the hypothalamic‐pituitary‐adrenal (HPA) axis and the consequently potentiated stress response have many adverse impacts on the brain and many other peripheral organs.^30^, ^31^ It has been demonstrated that hyperactivity of the HPA axis leads to the depressive behaviours mainly by elevating blood cortisol (CORT) levels and facilitating brain corticotropin‐releasing factor transmission.^32^ Hyperactivity of stress hormones can cause atrophy or apoptosis of neurons and decrease nerve regeneration in the hippocampus, amygdala and other depression‐related brain regions. There is growing evidence that neuroendocrine functional disorder might be the physiological basis of pathological changes of depression and other mood disorders.^33^ Researches over the last couple of years indicated that Rg1 could return the HPA axis to normal function, by improving serum CORT as well as testosterone levels and modulating glucocorticoid receptor (GR) levels in both prefrontal cortex (PFC) and hippocampus in rats with the CUMS‐induced depression.^34^ Ginsenoside Rh1 and Rg3 as oestrogen receptor (ERs) ligands and Rh2 and K as GRs ligands may also affect the function of HPA axis mediated by ERs or GRs.^35^ Furthermore, Rb1 and Rg3 could attenuate neuronal toxicity, which is related to glucocorticoid activity.^36^ In a study of CORT‐induced depression in mice, researchers found that ginseng total saponins (GTS) treatments improved depressant behaviours without changing the heightened serum CORT levels, as well as increased the downregulated levels of inhibitory GSK‐3β phosphorylation in the hippocampus, which was opposite to fluoxetine.^37^ In addition, studies have shown that some disorders of the gonadal hormones, such as oestrogen and progesterone, might affect feelings, emotions, and cognition which involved in the development of depression. There is strong evidence that menopause is a time of increased vulnerability to dysphoric mood.^38^ Moreover, in depressed patients, the HPG axis that regulates gonadal function was weakened.^39^ Rg1 showed antidepressant‐like effects not only by regulating the function of the HPA axis, but also the HPG axis. As is known, the level of androgen determines the androgen receptor (AR) expression, and Rg1 possessed an androgen‐like effect. It alleviated sleep disruption, decreased the CORT level in serum and increased the androgen receptor (AR) protein content without affecting testosterone level of the PFC of gonadectomized mice.^34^

### 3.3 Effect on BDNF

Brain‐derived neurotrophic factor (BDNF), one of the neurotrophic factors, does main favour in neurogenesis, neural survival and growth.^40^, ^41^ BDNF binds to its tropomyosin receptor kinase B (TrkB) to activate downstream signalling pathways and then completes phosphorylation and activation of the cAMP response element‐binding protein (CREB).^42^ The BDNF reduction in both hippocampus and PFC is closely associated with depression. Besides, the serum BDNF level also decreases in depression patients.^43^, ^44^ BDNF has acted as a biomarker for depression in many studies.^45^, ^46^, ^47^ The previous study illustrated that the extracts from Panax ginseng produce antidepressant‐like effect via accommodation of BDNF expression in various classic animal depression models, including FST, tail suspension test (TST), CMS, CUMS and so on.^48^, ^49^, ^50^ It has been reported that ginseng extract G115 could notably improve BDNF expression in the hippocampus and PFC of ethanol‐induced depression mice, even superior to amitriptyline, indicating that the antidepressant effects may contribute to some BDNF‐related signalling pathway.^51^ Interestingly, G115 did not influence the BDNF levels in normal brain areas. GTS could reverse alterations in depressive behaviours resulted from CORT through intervening in GSK‐3 β‐CREB signalling pathway in the hippocampus and reversing the reduction of some proteins related to plasticity.^52^ In the lipopolysaccharide (LPS) immunoreactive mice model which act as an inflammatory‐related animal model of depression, levels of BDNF and TrkB in the hippocampus were upregulated by sesquiterpenoids, suggesting that BDNF/TrkB pathway may be a target of the antidepressant effects of sesquiterpenoids.^53^ In addition, ginseng pectin also showed the antidepressant effect to some extent,^54^ which may be related to its neuroprotective role through the activation of ERK/MAPK and Akt survival signalling pathways.^55^

Ginsenoside monomers could similarly lead to apparent physiological behaviour changes in depressed murine. Ginsenoside Rg1 enhanced hippocampal BDNF signalling pathway while reduced serum CORT level, and it also reversed the decline in both dendritic spine density and hippocampal neurogenesis in the CMS model.^56^ Moreover, Rg1 could increase CREB phosphorylation and BDNF expression in the amygdala of CUMS rats.^57^ Downregulation of the Akt/mTOR signalling pathway has been also shown to be associated with decreased senescence of neural stem cells induced by ginsenoside Rg1.^58^ However, Rg1 seemed to have no distinguishable effect on the monoaminergic system.^56^ The same as sesquiterpenoids, ginsenoside Rg2 and Rg5 regulated the BDNF/TrkB pathway, which was confirmed by the blocking effect of TrkB shRNA and TrkB inhibitor.^59^, ^60^ Beyond this, researches showed that infusion of the BDNF signalling inhibitor blocked the antidepressant effects of Rg3.^61^

### 3.4 Effect on the neuroimmune system

Increasing evidence has indicated that inflammatory cytokine disorders may be closely related to the pathogenesis of depression. Some patients with MDD showed the main characteristics of inflammatory cytokine disorders in peripheral blood and cerebrospinal fluid. LPS is an effective and potent inducer of inflammatory cytokines (eg, TNF‐α; IL‐1β; IL‐6) that can acutely activate the innate immune system in the peripheral or central nervous system to induce depression‐like behaviours. Therefore, the LPS‐induced depression animal model was used to study the relationship between the immune system and depression. Accordingly, anti‐inflammatory therapy is becoming an effective treatment for depression and other neurological diseases. The antidepressant role of GTS is basically related to its anti‐inflammatory actions. It was found that GTS treatment can alleviate depression‐like behaviour impairments and decrease mRNA levels of IL‐1b, IL‐6, TNF‐a and IDO in the hippocampus. Reduced generation of various proinflammatory cytokines was detected in both LPS‐challenged mice and RAW264.7 cells after GTS treatment.^62^ Panax ginseng extract was found to reduce serum ACTH and CORT concentration, enhance Nrf2 and HO‐1 activities and inhibit inflammatory activity in the amygdala of chronic‐restraint stress (CRS)‐induced depressive mice, which resulted in enhanced BDNF activity and ultimately improved depressant‐like behaviours.^63^ Although ginsenoside Rg1, a cognized anti‐inflammatory agent, could not act directly in the brain, it was able to effectively mitigate the neuroimmune disturbances and neurochemical disturbances induced by central LPS challenge.^64^ Rg1 could significantly inhibit the expression of NADPH oxidase in frontal cortex and hippocampus of CRS‐induced cognitive impairment mice, thereby reducing the production of reactive oxygen species and reducing the oxidative damage of neurons in frontal cortex and hippocampal CA1 region.^65^ The inhibition of Nod‐like receptor pyrin domain‐containing protein 1 inflammasomes was also involved in the mechanisms underlying the effect of Rg1 on the glucocorticoid‐induced neuronal injury.^66^ In addition, ginsenoside Rg1 plays an important role in activating PPAR signalling to prevent apoptosis and inflammation.^67^, ^68^ Ginsenoside Rg3 improved depression‐like behaviours induced by systemic inflammatory via suppressing neuroinflammation and modulating TRP‐KYN metabolism.^69^ All these certified that targeting the peripheral immune system may work as a new therapy to neuropsychiatric disorders caused by neuroinflammation.

**Figure 1:**
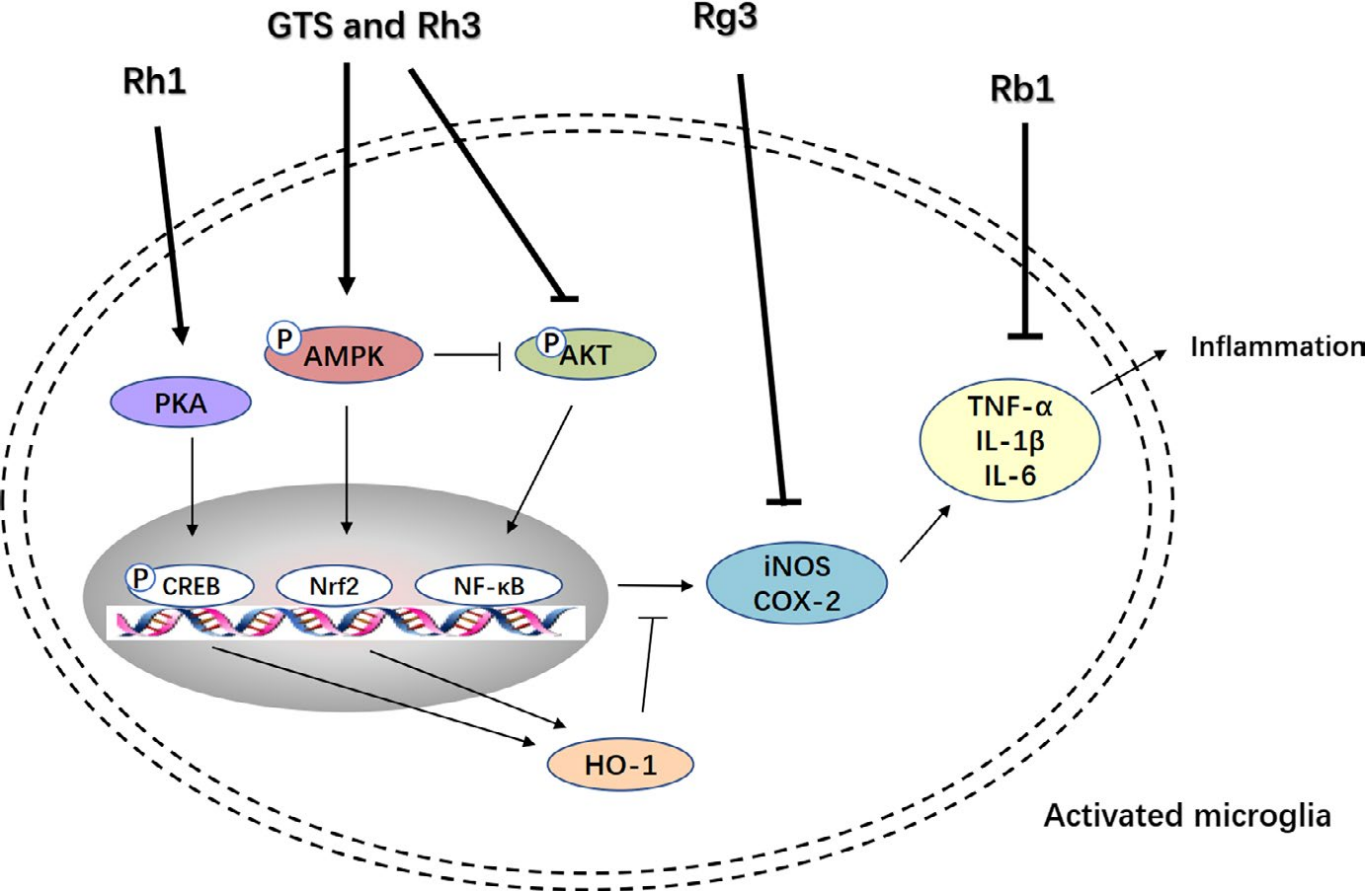
The action of ginsenosides on microglia. Akt, protein kinase B; AMPK, adenosine 5'‐monophosphate‐activated protein kinase; COX‐2, cyclooxygenase‐2; HO‐1, hemeoxygenase‐1; IL, interleukin; iNOS, inducible nitric oxide synthase; LPS, lipopolysaccharide; Nrf2, nuclear factor E2‐related factor 2; PI3K, phosphatidylinositol 3‐kinase; SIRT1, Sirtuin 1; TNF‐α, tumour necrosis factor‐α

Microglia, acting as cellular effectors of innate immunity, along with astrocytes, can drive the process of neuroinflammation by phagocytosis and cytokine release.^70^ An increasing body of evidence has validated the relationship between glial cells and neurological diseases. GTS was able to reverse the decrease of astrocyte number, structural changes and hippocampal atrophy in CORT‐induced depressed mice.^71^ Many studies have shown that systemic LPS administration immediately activates microglia in the brain, and this effect could be inhibited by ginsenosides. The effects of GTS and Rh3 were partly achieved by regulating 5'‐adenosine monophosphate‐activated protein kinase and its downstream signalling molecules, such as PI3K/Akt and NF‐κB/ nuclear factor E2‐related factor 2, thereby inhibiting the expression of inducible nitric oxide synthase and proinflammatory cytokines and enhancing the expression of anti‐inflammatory hemeoxygenase‐1.^72^, ^73^ The regulatory role of Rh1 is based on the physiological basis of regulating inflammation, neurotoxicity and oxidation through PKA and hemeoxygenase‐1.^74^ Rg3 also decreased the expression of inducible nitric oxide synthase, cyclooxygenase‐2, TNF‐α, IL‐1β and IL‐6 in brain tissue after LPS injection.^75^ Moreover, Rb1 attenuates damage to cerebral cortex neurons by downregulation of nitric oxide, superoxide and TNF‐α expression in hypoxia‐activated microglia.^76^ These studies may provide therapeutic potential for the treatment of depression with microglia activation (Figure 1).

### 3.5 Effect on other mechanisms

Panax quinquefolium has shown the protective effects against olfactory bulbectomy‐induced depression‐like behaviours and nitric oxide pathway might be involved.^77^ GTS, ginsenoside Rg3 and Rb1 were demonstrated to resist stress by blockage of the activity of ODC and reduction of the accumulation of PUT, suggesting that they have potential neuroprotective effects in the brain.^78^ It was found that the water extract of Panax ginseng could protect cells from CORT‐induced impairments via the intervention of GR‐related functional proteins such as histone deacetylase 6 and heat‐shock protein 90 in vitro and succedent functional recovery of endoplasmic reticulum and mitochondria.^79^ In vitro, Rg3 recovered growth and suppressed apoptosis by regulating the cell cycle in HT22 cells treated by *N*‐methyl‐d‐aspartic acid.^80^ Amyloid‐β peptides (Aβ), regarded as a main pathogenic factor of Alzheimer's disease, is playing an emerging role in stress response as well as in depression. Aβ administration in rats induced depression‐like behaviours, changed the activation of the HPA axis and decreased the levels of 5‐HT and neurotrophic factors in the cortex.^81^ Rg2 might protect PC12 cells from Aβ25‐35‐induced apoptosis by affecting the PI3K/Akt signalling pathway.^82^ Moreover, numerous reports have shown that many other kinds of ginsenosides, such as Re, Rg1 and Rf, had the capability to downregulate the Aβ deposition and the tau protein phosphorylation.^83^, ^84^, ^85^ These results suggest that these ginsenosides might have antidepressant effects through regulating Aβ. It was known that gap junction intercellular communication (GJIC) dysfunction might be involved in the potential pathogenesis of depression.^86^ Pre‐treatment with Rg1 can ameliorate GJIC in the astrocytes of the PFC and hippocampus in rats treated with CORT. Also, the Cx43 expression was increased and the Cx43 phosphorylation was decreased, which may be of great significance for the treatment of depression.^87^, ^88^

**Figure 2:**
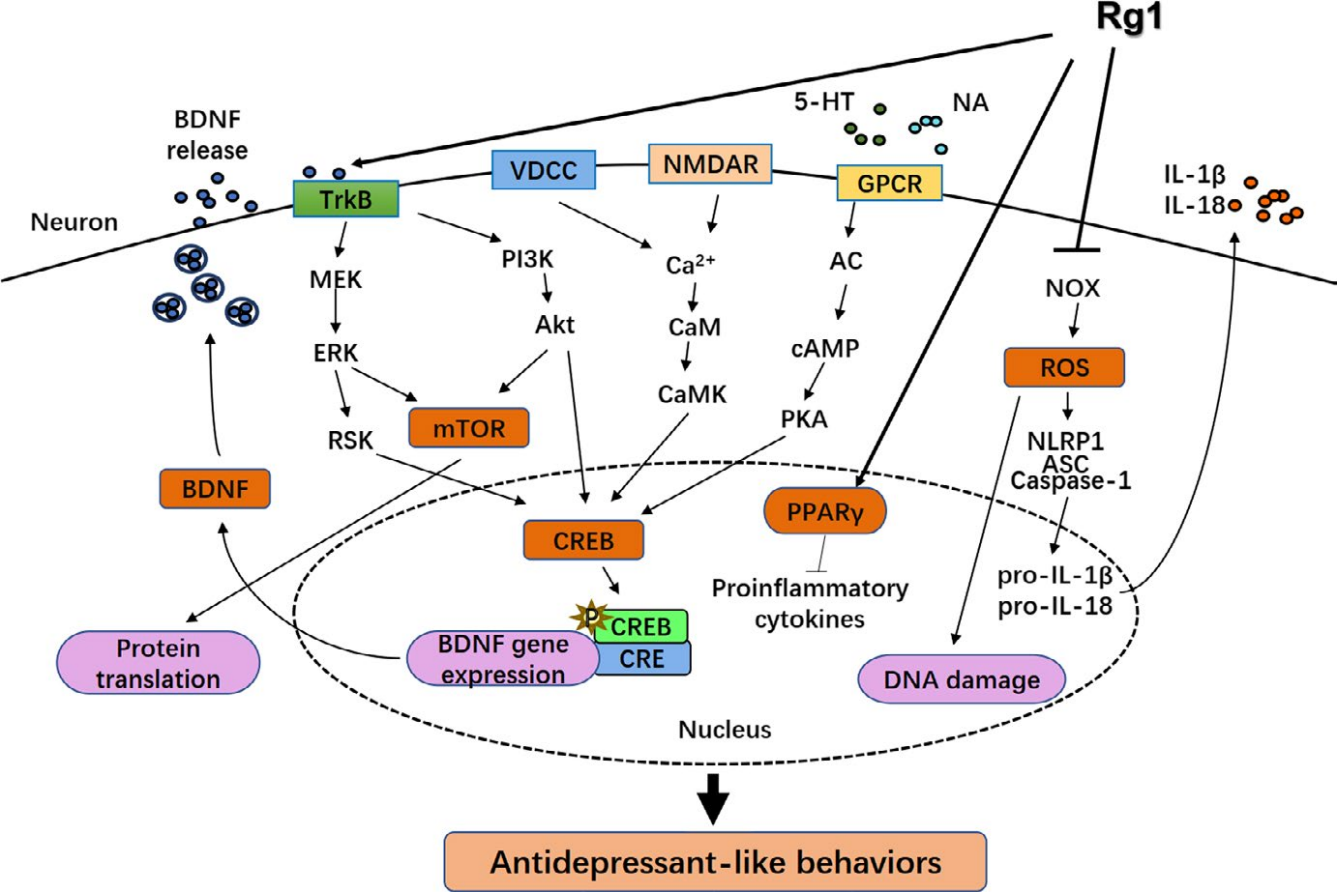
The antidepressant action of ginsenoside Rg1. 5‐HT, 5‐hydroxytryptamine; Akt, protein kinase B; ASC, apoptosis‐associated speck‐like protein containing CARD; BDNF, brain‐derived neurotrophic factor; CaM, calmodulin; CaMK, CaM kinases; cAMP, cyclic adenosine monophosphate; caspase‐1, cysteinyl aspartate specific proteinase‐1; CRE, cAMP response element; CREB, cAMP response element‐binding protein; ERK, extracellular signal‐regulated kinase; GPCR, guanosine‐binding protein‐coupled receptor; IL, interleukin; MEK, MAP/ERK kinase; mTOR, mechanistic target of rapamycin; NA, noradrenaline; NLRP1, Nod‐like receptor pyrin domain‐containing protein 1; NMDAR, N‐methyl‐D‐aspartate receptor; NOX, NADPH oxidase; PI3K, phosphatidylinositol 3‐kinase; PKA, protein kinase A; PPARγ, peroxisome proliferator‐activated receptor γ; ROS, reactive oxygen species; RSK, ribosomal S‐6 kinase; TrkB, tropomyosin‐related kinase B; VDCC, voltage‐dependent calcium channel

Overall, many studies have shown that the regulatory role of Panax ginseng appears to be crucial in depression (Table 4). The action of Rg1 is shown in Figure 2 as an example of intracellular signalling pathways that illustrates the antidepressant action of ginseng active ingredients. Considering that Panax ginseng and its active ingredients were reported to regulate the monoamine neurotransmitters, HPA axis, neurotrophic factors and neurogenesis, the relationship between Panax ginseng and the pathogenesis of depression might be a potential novel target for depression treatment.

**Table 4:** Pharmacological activity and mechanism of Panax ginseng on depression in experimental models

No.	Ingredients	Medication dosage	Subjects	Depression models	Behaviour tests	Mechanisms	Refs.
1	Water extract	6.25, 12.5, 25, 50, 100, 200 μg/mL	PC12 cells	CORT‐induced apoptosis	—	Possesses neuroprotection in PC12 cells by the intervention of HDAC6 and HSP90 of the GR‐related function proteins and subsequent restoration of ER and mitochondria functions	79
		75, 150, 300 mg/kg	Mice	CRS	FST, TST	Suppresses neuroinflammatory response and upregulates Nrf2 signalling in the amygdale	63
2	Extract G115	100, 200, 400, 800 mg/kg	Mice	Ethanol treatment	OFT, FST	Increases BDNF levels in the hippocampus and PFC	51
3	Ginseng total saponins	100 mg/kg	Rats	CUMS	OFT, SPT	Affects ACTH, CORT and attenuates alterations in catecholamines and 5‐HT metabolites in cerebrum and peripheral areas	23
		10, 30, 100, 300, 1000 mg/kg	Rats	CMS	OFT, clonidine aggression, 5‐HTP head‐twitch test	Increases the levels of 5‐HT, DA and NA in brain and reduces intracellular Ca^2+^ concentration	29
		12.5, 25, 50 mg/kg	Mice	CORT	FST, TST	Raises the downregulated levels of hippocampal GSK‐3β inhibitory phosphorylation without altering the elevated serum CORT levels	37, 52
		25, 50, 100 mg/kg	Rats	CMS	SPT, OFT, NIH test	Enhances the NE, DA, DOPAC and HVA levels and BDNF expression in the hippocampus	48
		1, 5, 20, 100 μg/mL 200 mg/kg	RAW264.7 cells Mice	LPS treatment LPS treatment	— OFT, SPT, FST, TST	Decreases production of various proinflammatory cytokines in both LPS‐challenged mice and RAW264.7 cells	62
		1, 5, 25, 50 mg/kg	Mice	CORT	FST, TST	Protects hippocampal astrocyte structural plasticity through reversing the decrease of astrocyte number, structural changes, and hippocampal atrophy	71
		5, 10, 50, 100 μg/mL 30, 50 mg/kg	BV2 cells and B35 cells Mice	LPS treatment LPS treatment	—	Suppresses microglial activation through significantly suppressed NF‐κB and MAPK activities, which inhibited expression of inflammatory signalling molecules like iNOS, MMP‐9 and proinflammatory cytokines	72
4	Gintonin	0.01‐1 µg/mL 50, 100, 200 mg/kg	BON cells Mice	— AWS	— FST, TST	Attenuates depressive‐like behaviours by mediating 5‐HT release from intestinal enterochromaffin cells	28
5	Sesquiterpenoids	0.25, 1 mg/kg	Mice	LPS‐induced depression	FST, TST	Neurotrophy and anti‐inflammatory defences through the BDNF/TrkB and Sirt 1/NF‐κB signalling pathways	53
6	Rb1	4, 8, 16 mg/kg	Rats	CUMS	OFT, FST	Upregulates the central monoamine neurotransmitters (5‐HT, 5‐HIAA, NA and DA levels)	26
		2.5, 5, 10 mg/kg	Mice	OVX	FST, GMA, MUW	Increases 5‐HT_2A_ receptor binding	27
		1, 5, 10 μmol/L	SHSY‐5Y cells and rat brain OHSCs	DEX treatment	—	Targets GC action through downregulation of pro‐apoptotic bax expression, neuronal death in hippocampal slice and ROS generation	36
		100 μg/mL	N9 microglial cells Rats	Hypoxia exposure	—	Attenuates damage to cerebral cortex neurons by downregulation of nitric oxide, superoxide and TNF‐α expression in hypoxia‐activated microglia	76
7	Rg1	5, 10, 20, 40 mg/kg	Mice	CUMS and GDX	FST, TST, SPT, OFT, MPS	Improves serum CORT and testosterone levels and modulates the expression of GR and AR to recover the HPA and HPG axis to normal function	34
		40 mg/kg	Rats	CUMS	SPT, FST	Activates CREB/BDNF signalling pathway in the PFC, amygdala and BLA	49, 50, 57
		2.5, 5, 10, 20 mg/kg	Mice	CMS	FST, TST	Activates the BDNF‐TrkB signalling pathway via increased levels of pERK1/2 and pCREB and promotes neurogenesis in the hippocampus	56
		20 μg/mL 20 mg/kg	NSCs Mice	d‐gal d‐gal	— MWM	Decreases the level of oxidative stress and the phosphorylation levels of Akt and mTOR thus inhibiting NSCs ageing	58
		10, 30 mg/kg	Rats	Central injection of LPS	Weight loss, food consumption, SPT	Dampens microglial activation, inhibits IL‐1β, IL‐6 and TNF‐α mRNA levels and neurotoxic species in the central compartment via peripheral regulatory effects	64
		2, 5 mg/kg	Mice	CRS	MWM	Decreases ROS generation and alleviates the neuronal oxidative damage in the frontal cortex and hippocampus CA1	65
		1, 2, 4 mg/kg	Mice	CRS	OFT, NOR test	Increases the expression of GR and decreases the expression of NLRP 1, ASC, caspase 1, caspase 5, IL‐1β and IL‐18 in the hippocampus	66
		20, 40 mg/kg	Rats	MCAO	—	Inhibits hippocampal cell apoptosis and inflammation by regulating PPARγ/HO‐1 signalling pathway	67
		30, 60 mg/kg	Rats Primary cortical neurons	MCAO OGD	—	Upregulates PPARγ expression	68
8	Rg2	10, 20 mg/kg	Mice	CMS	FST, TST, OFT, SPT	Promotes the hippocampal BDNF signalling pathway	59
		5, 10, 20 μg/mL	PC12 cells	Aβ25‐35‐induced apoptosis	—	Protects against Aβ25‐35‐induced apoptosis in PC12 cells via upregulation of Bcl‐2 and downregulation of Bax, two critical downstream effectors in PI3K/Akt signalling	82
9	Rg3	1, 5, 10, 20 μmol/L	SHSY‐5Y cells and rat brain OHSCs	DEX treatment	—	Targets GC action through downregulation of pro‐apoptotic bax expression, neuronal death in hippocampal slice and ROS generation	36
		10, 20 mg/kg	Mice	CSDS	FST, TST, OFT	Promotes of the hippocampal BDNF signalling pathway	61
		1, 5, 10 μmol/L	HT22 cells Mice	NMDA treatment CMS	— FST, TST, SPT	Recovers proliferation and inhibits apoptosis by altering the cell cycle Promotes the phosphorylation of CREB and BDNF signalling in the hippocampus	80
		20, 40 mg/kg	Mice	LPS‐induced depression	OFT, FST, TST	Suppresses IL‐1β, IL‐6, TNF‐α and IDO disorders and modulates TRP‐KYN metabolism both in the brain and in the periphery	69
		10, 20, 30 mg/kg	Mice	LPS treatment	—	Attenuates microglia activation through decreasing the expression of iNOS, COX‐2, TNF‐α, IL‐1β and IL‐6 in brain tissue	75
10	Rg5	5, 10, 20, 40 mg/kg	Mice	CSDS	FST, TST, OFT	Activation of hippocampal BDNF signalling cascade	60
11	Rh1	0, 100, 300 μmol/L 50 mg/kg	Microglia Mice	LPS stimulation LPS treatment	— —	Activates PKA and its downstream effector, HO‐1, to modulate pro‐ and anti‐inflammatory molecules in activated microglia	74
12	Rh3	30, 50 μmol/L	BV2 cells 2 Rat primary microglia	LPS treatment	—	Regulates AMPK and its downstream signalling molecules, such as PI3K/Akt and NF‐κB/ Nrf2, thereby inhibiting the expression of iNOS and proinflammatory cytokines and enhancing the expression of anti‐inflammatory HO‐1	73

Abbreviations: 5‐HT_2A_R: 5‐HT_2A_ receptors; ACTH: adrenocorticotropic hormone; Akt, protein kinase B; AMPK, adenosine 5'‐monophosphate‐activated protein kinase; AR: androgen receptor; AWS: alcoholic withdrawal syndromes; Aβ: amyloid‐β peptides; BDNF: brain‐derived neurotrophic factor; BW: body weight; CORT: corticosterone; COX‐2, cyclooxygenase‐2; CRS: chronic‐restraint stress; CSDS: chronic social defeat stress; CUMS: chronic unpredictable mild stress; CUS: chronic unpredictable stress; DEX: dexamethasone; D‐gal: D‐galactose, DA: dopamine; DOPAC: 3,4‐hydroxyphenylacetic acid; EPM: elevated plus‐maze; FC: food consumption; FST: forced swimming test; GMA: general motor activity; GR: glucocorticoid receptor; HDAC6: histone deacetylase 6; HO‐1, hemeoxygenase‐1; Hsp90: heat‐shock protein 90; HVA: homovanillic acid; IDO: indoleamine‐2,3‐dioxygenase; IL: interleukin; iNOS, inducible nitric oxide synthase; IκB‐α: inhibitor of κB‐α; KYN: kynurenine; LPS: lipopolysaccharide; MAPK, mitogen‐activated protein kinase; MCAO: middle cerebral artery occlusion; MPS: measurement of pentobarbital‐induced sleep; mTOR, mechanistic target of rapamycin; MUW: measurement of uterine weight; MWM: Morris water maze; NA: noradrenaline; NF‐κB: nuclear factor‐κB; NIH test: novelty‐induced hypophagia test; NOR: novel object recognition; Nrf2, nuclear factor E2‐related factor 2; NSCs: neural stem cells; OFT: open field test; OGD: oxygen glucose deprivation; OHSC: organotypic hippocampal slice cultures; OVX: ovariectomy; PI3K, phosphatidylinositol 3‐kinase; PPARγ, peroxisome proliferator‐activated receptor γ; SA: spontaneous activity; Sirt 1: Sirtuin type 1; SIT: social interaction test; SPT: sucrose preference test; TNF: tumour necrosis factor; TST: tail suspension test.

## 4 ANTIDEPRESSANT EFFECTS OF REPRESENTATIVE PANAX GINSENG HERBAL FORMULAE

### 4.1 Kai Xin San

Kai Xin San (KXS) is a classical Chinese herbal prescription which is composed of four kinds of medicines: Panax ginseng, Polygala tenuifolia, Poria Cocos and Acorus calamus. As a classic formula still used today, KXS should benefit Qi first; therefore, ginseng is the main medicine in the prescription. Only when ginseng invigorating Qi and cultivating blood, can KXS achieve the effect of calming the mind. Therefore, the compatibility features of the prescription are that Panax ginseng and Poria cocos are used to tonify Qi and nourish the heart, combined with Polygala and calamus to tranquilize the mind. Clinically, the compatibility ratio of KXS varies according to the specific situation, which results in the serial formulations of KXS.

Chemical studies showed that the main components of KXS were ginsenosides, Polygala tenuifolia glycosides and volatile oils of Acorus calamus. It is through these main active substances that KXS exerts neuroprotective and antidepressant effects. Dang et al found that KXS can reduce the expression of AChE in the hippocampus, increase concentrations of various monoamine neurotransmitters in the hippocampus and PFC and reduce the concentration of serum ACTH, significantly improving the depression symptoms in mice subjected to CMS.^89^ KXS was found to ameliorate 5‐HT defects, which is achieved by raising the synthesis, inhibiting the reuptake and ultimately increasing the concentration of intracephalic 5‐HT.^90^, ^91^ In an earlier study, impacts and potential mechanisms of KXS2012 on depression in both cell and animal models were validated. Moreover, it promoted neurogenesis via inducing the expression of synaptotagmin and dendritic spine density in cultured neurons and increased neurotrophins in astrocytes which might concern the regulation of Erk1/2 and CREB phosphorylation.^92^ In further studies of Zhu et al,^93^ KXS was confirmed to promote expressions of neurotrophic factors and TrkB receptors. By combining qPCR, Western blotting and constituent identification analysis, ginsenoside Rg1 and Rb1 may be active compounds in this component that increase NGF and BDNF expression by activating cAMP‐dependent signalling pathway and stimulating neurotrophic factor synthase.^94^ Studies both in vivo and vitro have shown that KXS can facilitate hippocampal synaptogenesis by upregulating synaptic proteins expression.^95^ There is also evidence that KXS seems to improve memory impairment induced by hindlimb suspension mainly by decreasing serum ROS, 8‐OHdG and 3‐nitrotyrosine and inhibiting oxidative stress injury, rather than influencing the central cholinergic system.^96^ In addition, KXS treatment effectively improved depressive symptoms in fluoxetine‐resistant depression rats by the reduction of COX‐2, IL‐2, IL‐6, TNF‐α levels and increase of IL‐10, IFN‐γ level.^97^ With the deepening of research, the neurobiological mechanism of KXS and its specific active ingredients in preventing and treating depression will be further elucidated.

### 4.2 Shen Yuan Gan

Ginseng and Polygala tenuifolia are often used together in the clinical treatment of depression and have shown good antidepressant effects in animal experiments and clinical studies. Shen Yuan Gan (SYG), a Chinese herbal compound, consists of GTS and Polygala tenuifolia total glycosides (PTG) in a proportion of 2 to 1. Literature reports showed that the effective components of Panax ginseng and Polygala tenuifolia against depression were GTS and PTG, respectively. The antidepressant effects of SYG have been confirmed in various depression models. In previous studies, Sun et al^98^ found that SYG administration could alleviate depression in TST and FST, as well as regulate CORT levels of learned helplessness rats and BDNF levels in the hippocampus of CMS rats. In subsequent trials, Sun et al^99^ found SYG not only ameliorated CMS‐induced changes, but also recovered concentrations of 5‐HT, DA, NA and acetylcholine (Ach) in the PFC, which indicated that antidepressant actions of SYG are likely to be connected with the level of monoamine neurotransmitters. Besides, SYG may also enhance cognition by increasing ACh in the PFC. Therefore, SYG has a clear antidepressant effect and can be used as a potential selective antidepressant.

### 4.3 Xiaochaihu Decoction

Xiaochaihu Decoction (XD), originated from Shanghan Zabing Lun of Zhang Zhongjing‐a distinguished physician in Eastern Han Dynasty, has been used in the treatment of Shaoyang syndrome which involves depressive‐like symptoms in China for thousands of years. After many years of evolution, the prescription of XD has the merits of strict compatibility and definite curative effect. It is composed of seven herbs: Bupleurum as the monarch drug, Scutellaria as the minister drug, Panax ginseng, Pinellia and Glycyrrhiza as the assistant drug and Ginger and Jujube as the guide drug.^100^ Among them, Scutellaria, Panax ginseng and Glycyrrhiza might be the core components of XD, which has the function of strengthening the spirit and tonifying Qi. Clinical and modern pharmacological studies of XD have proved that it has a satisfactory antidepressant effect, and the active ingredients are mainly flavonoids and saponins.

Lots of animal researches have proven that the therapeutic effects of XD on depressant‐like behaviours are likely mediated by upregulating the monoamine neurotransmitter, neurotrophin expression and neurogenesis in related brain regions of rodents. Su et al^101^ found that XD improved depressant‐like behaviours, including TST, FST, hypothermia induced by reserpine and head‐twitch induced by 5‐HTP, which may be related to the increased levels of 5‐HT, 5‐hydroxyindoleacetic acid and 5‐HT turnover in the hippocampus and PFC. In their further study, the 5‐HT and DA concentrations, and the BDNF, NGF, TrkB and TrkA expressions in the hippocampus also significantly increased after XD administration, which was reduced in CUMS rats.^102^ Moreover, XD treatment in chronically isolated rats might increase neurogenesis by upregulating 5‐HT levels, activating the 5‐HT_1A_ receptor and then promoting expressions of downstream CREB and BDNF in the hippocampus.^103^ XD could remarkably alleviate chronic CORT‐induced anxiety/depression‐like behaviours, which were probably attributed to promoting hippocampal neurogenesis and remodelling the integrity of the negative feedback loop on the HPA axis.^104^ Additionally, XD was found for the first time to enhance the expression of monoamine neurotransmitter synthase (TPH2 and TH), inhibit the expression of 5‐HT transporter and decrease the expression of monoamine neurotransmitter degrading enzyme (MAOA) in the hippocampus of social isolation‐reared mice.^105^ These studies lay the foundation for the study of pharmacodynamic substances of XD's antidepressant effect.

As mentioned above, Panax ginseng herbal formulae have multiple functions on anti‐depression and the confirmation of the mechanisms is progressing (Table 5). For the classical prescriptions used so far, there are few systematic studies on their specific effective material basis for the treatment of depression. In recent years, in the study of the modernization of traditional Chinese medicine, it is a hot spot to take the traditional Chinese formulae as the natural combinatorial chemical library and to study it by the method of combinatorial Chinese medicine. That is to say, by separating and extracting every single drug in the compound, the specific effective molecules of traditional Chinese medicine are combined together to synthesize a relatively safe natural combinatorial chemical library with curative effect. It is also the direction for further systematic research.

**Table 5:** Compositions and antidepressant effects of Panax ginseng herbal formulae

Formulae	Composition	Active ingredients	Antidepressant effects	Refs.
KXS	Panax ginseng Polygala Tenuifolia Poria cocos Acorus calamus	Ginsenosides, Polygala tenuifolia glycosides, Volatile oils of Acorus calamus	Modulates the HPA axis and levels of 5‐HT, DA, NE and AChE	89, 90, 92
			Increases the protein levels of BDNF, NGF and GDNF, as well as the mRNA expressions of Trk receptors	92, 93, 94
			Promotes neurogenesis and synaptogenesis	92, 95
			Influences the inflammatory processes via reduction of COX‐2, IL‐2, IL‐6, TNF‐α levels and increase of IL‐10, IFN‐γ levels	97
SYG	GTS, PTG	GTS, PTG	Modulates the levels of 5‐HT, DA, NE and ACh	99
XD	Bupleurum, Scutellaria, Panax ginseng, Pinellia, Ginger, Glycyrrhiza, Jujube	Flavonoids Saponins	Regulates the serotonergic system and DA level	101, 102, 103, 105
			Improves the BDNF, NGF, TrkB and TrkA expressions	102, 103
			Facilitates neurogenesis and remodel the negative feedback loop on HPA axis	104

Abbreviation: ACh: acetylcholine; AChE: acetylcholinesterase; BDNF: brain‐derived neurotrophic factor; COX: cyclooxygenase; DA: dopamine; GDNF: glial cell‐derived neurotrophic factor; GTS: ginseng total saponins; IFN: interferon; IL, interleukin; KXS: Kai Xin San; NE: norepinephrine; NGF: nerve growth factor; PTG: polygala tenuifolia total glycosides; SYG: Shen Yuan Gan; TNF, tumour necrosis factor; Trk: tropomyosin receptor kinase; XD: Xiaochaihu Decoction.

## 5 EFFECTS OF PANAX GINSENG ON OTHER CNS DISEASES

Effects of Panax ginseng and its active ingredients on other neurological diseases, like Alzheimer's and Parkinson's, are well documented in several studies.^106^, ^107^ Since the NTFs are implicated in some other CNS disorders, extracts and natural products of Panax ginseng exhibit therapeutic effects on various kinds of disease models.^108^ It is possible that Rg3 could be used to treat these disorders based on the regulation of BDNF expression, which needs to be further studied.^61^ Furthermore, Rg1 could alleviate cognitive and sexual dysfunctions, which might be associated with depressive symptoms.^109^, ^110^ Ginsenosides Rg5 and Rh3 might inhibit AChE activity dose dependently and reverse the reduction of BDNF and phosphorylated CREB in the hippocampus, resulting in protection of memory deficit.^111^ American ginseng could play a protective role by regulating nitrergic signalling cascade to resist the cognitive impairment, neuroinflammation and biochemical alterations induced by chronic unpredictable stress.^112^

## 6 CONCLUSIONS

In conclusion, in this study we summarized the recent progress of antidepressant effects and underlying mechanisms of Panax ginseng and its representative herbal formulae. The molecular and cellular mechanisms of Panax ginseng and its herbal formulae include modulating monoamine neurotransmitter system, upregulating the expression of neurotrophic factors, regulating the function of HPA axis, and anti‐inflammatory action. This review provides theoretical bases and clinical applications for the treatment of depression by Panax ginseng and its representative herbal formulae.

## CONFLICTS OF INTEREST

The authors declare no conflict of interest.

## AUTHOR CONTRIBUTIONS

BL contributed conception and design of the study; YJ wrote the first draft of the manuscript; LZ and JF wrote sections of the manuscript; RC provided the critical revisions. All authors revised the manuscript and approved the submitted version.

## Data Availability

Research data are not shared.
